# Differentiating peripheral cholangiocarcinoma in stages T1N0M0 and T2N0M0 from hepatic hypovascular nodules using dynamic contrast-enhanced MRI

**DOI:** 10.1038/s41598-017-08634-2

**Published:** 2017-08-14

**Authors:** Shihong Li, Haizhen Qian, Yu Peng, Huihui Jia, Guangwu Lin

**Affiliations:** 1 Huadong Hospital Affiliated to Fudan University, Department of Radiology, Shanghai, 200040 China; 2 Affiliated Hospital of Jining Medical University, Department of Radiology, Jining, Shandong 272029 China

## Abstract

Because cholangiocarcinoma shows no specific clinical signs or symptoms and presents with non-specific biological and tumor markers in the early stages, MRI findings often lack typical features before this lesion becomes symptomatic and might be mistaken for other liver lesions. An evaluation of relevant radiological findings in nodular cholangiocarcinoma (≤3 cm) in stages T1N0M0 and T2N0M0 is urgently needed. In our study, we compared two groups of liver hypovascular nodules and found that a distinct margin and enhanced area/nodule size >2/3 in the delayed phase were more frequently observed in cholangiocarcinoma cases than in metastatic nodule cases in which markedly high signal intensity on T2WI was common (p < 0.05). The results also revealed that in the both the portal and delayed phases, an enhanced area/nodule size >2/3 favored cholangiocarcinoma, whereas the presence of regional markedly higher SI on T2WI favored benign nodules. Furthermore, signs of peripheral washout in the delayed phase only appeared in cholangiocarcinoma cases.

## Introduction

Although the overall prognosis of cholangiocarcinoma is generally poor, curative resection of early stage cholangiocarcinoma may improve the chances of long-term survival^1–3^. In clinical practice, the majority of cholangiocarcinomas present at an advanced stage; however, early stage cancers are incidentally diagnosed with increasing frequency. Therefore, treatment options for unexpected cancers have attracted significant attention^4^. However, preoperative diagnosis of early stage cholangiocarcinoma has not been studied as frequently.

Cholangiocarcinoma shows no specific clinical signs or symptoms and presents with non-specific biological and tumor markers in early stages of the disease^5^. Cross-sectional imaging plays an increasing role in the diagnosis of liver diseases because most common liver neoplasms can be characterized with the use of advanced imaging techniques. To detect early cases of cholangiocarcinoma that may present as a nodule, magnetic resonance imaging (MRI) is accepted as a powerful tool^6, 7^. Cholangiocarcinomas have been defined as hypovascular liver tumors as well as metastatic liver tumors, especially in comparison to colorectal or breast cancers and other benign nodules^8^. However, the imaging appearances of various nodular hypovascular entities overlap considerably. Nodular cholangiocarcinoma remains challenging due to a lack of typical findings, such as capsular retraction and dilatation of the peripheral intrahepatic ducts.

To our knowledge, an evaluation of the radiological findings in nodular cholangiocarcinoma (≤3 cm) in stages T1N0M0 and T2N0M0 has not been performed. Accordingly, we conducted this study to compare the diagnostic performance of nodular cholangiocarcinoma vs. that of other common hypovascular hepatic lesions (≤3.0 cm).

## Results

This study included 26 peripheral nodular cholangiocarcinomas (PCCs), 23 hypovascular metastases (HMs), 19 solitary necrotic nodules (SNNs) and 32 atypical hepatic hemangiomas (HGs). The mean diameter of the lesions examined was 1.8 ± 0.7 cm (range 0.5-3.0 cm, n = 100). The main imaging features are summarized in Tables 1 and 2 (Supplementary dataset 1).

**Table 1:** MRI findings of peripheral cholangiocarcinoma and metastatic hepatic hypovascular nodules.

MRI findings	PCC (26)	HM (23)	χ^2^ value	P
Location				
Subcapsular	9 (35)	13 (57)	2.367	0.124
Deep parts of lobe	17 (65)	10 (43)		
Margin				
Sharp	22 (85)	0	—	0.000
Indistinct	4 (15)	23 (100)		
Regionally markedly high SI on T2WI	3 (12)	13 (57)	9.277	0.002
Enhanced area/nodule size^a^				
Portal phase				
>2/3	9 (35)	3 (13)	2.015	0.156
Delayed phase				
>2/3	23 (88)	2 (9)	—	0.000
Peripheral washout sign	11 (42)	0	—	0.000

Note: a, Estimate at the maximum cross-sectional area. Data are presented as the number of patients with percentage in parentheses. Percentages are calculated on the basis of each group. PCC, peripheral cholangiocarcinoma. HM, hepatic metastases. SI, signal intensity.

**Table 2:** MRI findings of peripheral cholangiocarcinoma and benign hepatic hypovascular nodules.

MRI findings	PCC (26)	Benign nodules (51)		χ^2^ value	P
		SNNs (19)	HG (32)		
Location					
Subcapsular	9 (35)	10 (20)	17 (33)	2.323	0.127
Deep parts of lobe	17 (65)	9 (18)	15 (29)		
Margin					
Sharp	22 (85)	15 (29)	32 (63)	—	0.432
Indistinct	4 (15)	4 (8)	0		
Markedly high SI area on T2WI	3 (12)	7 (14)	32 (63)	26.724	0.000
Enhanced area/nodule size^a^					
Portal phase					
>2/3	9 (35)	2 (4)	4 (8)	5.733	0.017
Delayed phase					
>2/3	23 (88)	0	9 (18)	32.700	0.000
Peripheral washout sign	11 (42)	0	0	—	0.000

Note: a, Estimate at the maximum cross-sectional area. Data are presented as the number of patients with percentage in parentheses. Percentages are calculated on the basis of each group. SI, signal intensity. A difference with p < 0.05 was considered to be statistically significant for the Chi-square test, and the Fisher’s exact test was used to compare each MRI finding between the PCC and benign groups. PCC, peripheral cholangiocarcinoma. SNN, solitary necrotic nodules. HG, hepatic hemangioma. SI, signal intensity.

**Figure 1:**
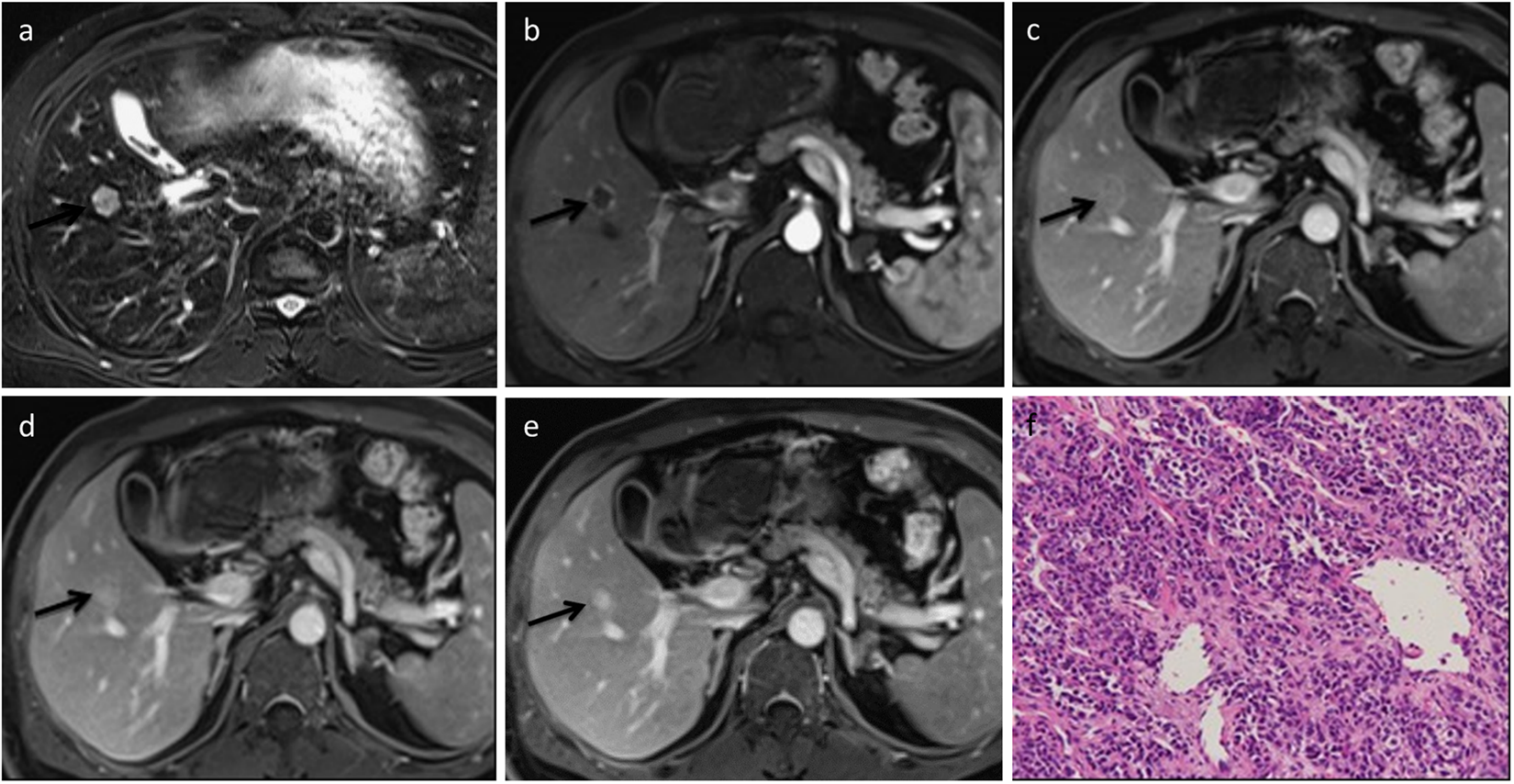
Peripheral nodule cholangiocarcinoma in a 68-year-old man. (**a**) MRI of the nodule in segment V, approximately 17 mm in diameter, by transverse T2WI; the nodule is slightly hyperintense relative to that of the liver parenchyma. (**b**) The arterial phase shows that the nodule has no hyperintense portion compared with that of the liver parenchyma except for peripheral rim-like enhancement. (**c**) Enhanced area/nodule size >2/3 in the portal phase. (**d**,**e**) Delayed phase (3 and 5 min) images clearly demonstrate cloud-like contrast filling with a low-SI peripheral rim. (**f**) Histological examination was diagnostic of cholangiocarcinoma (hematoxylin and eosin-stained section; original magnification, 200×).

**Figure 2:**
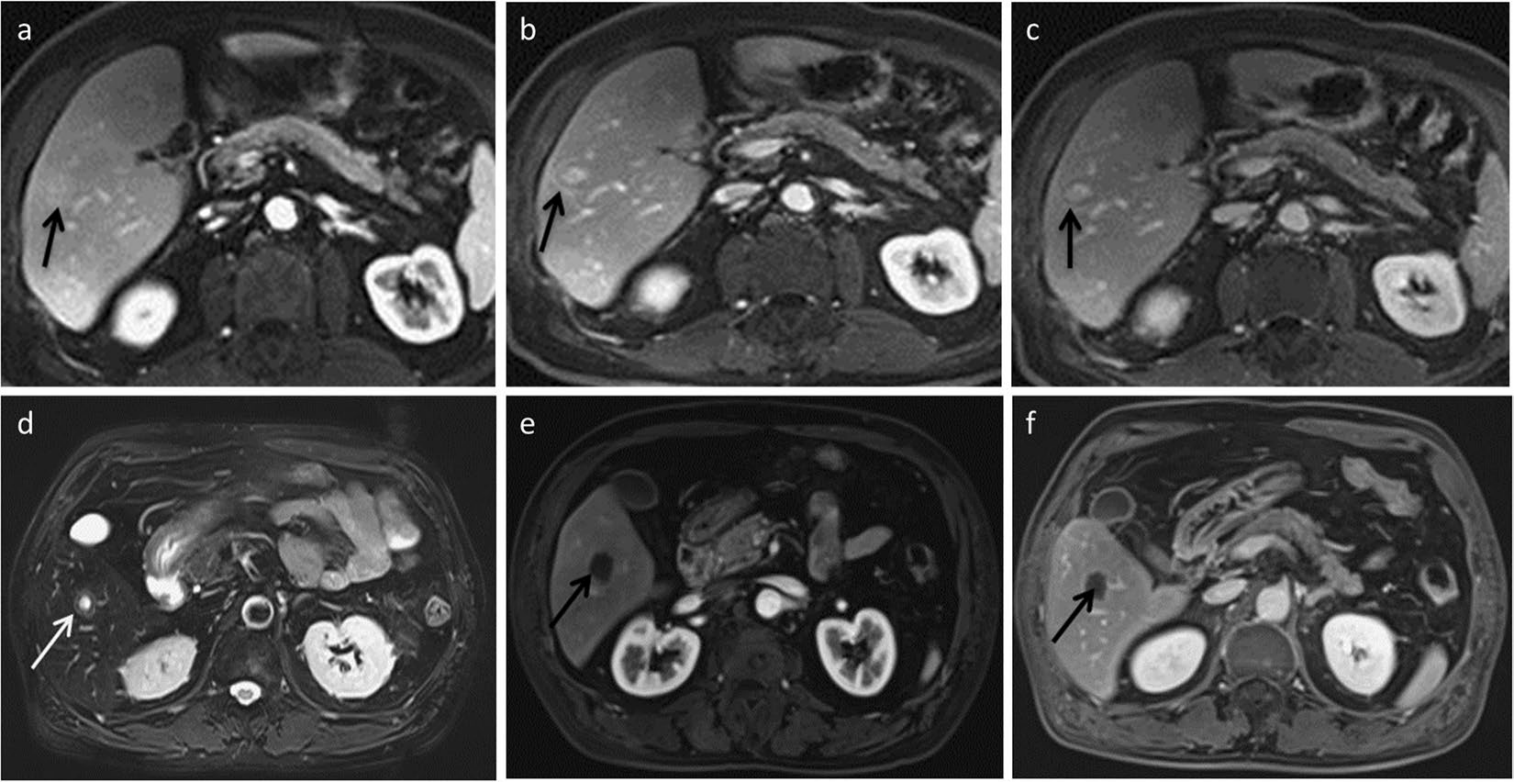
Two cases of hypovascular nodules. (**a**–**c**) Peripheral nodule cholangiocarcinoma in a 57-year-old man. (**a**) MRI of the nodule in SV, approximately 22 mm in diameter; the nodule was indistinct with an enhanced area/nodule size >2/3 in the portal phase. (**b**,**c**) Delayed phase (3 and 5 min) images clearly demonstrate signs of peripheral washout, which was histopathologically proven to be a cholangiocarcinoma. (**d–f**) Solitary necrotic nodule in a 63-year-old man. On transversal fat-saturated T2WI (**d**), the center of the nodule is hyperintense relative to the liver parenchyma, but the ring-like margin of the nodule is relatively slightly hyperintense. The (**e**) arterial and (**f**) delayed phases show that the nodule has no significantly enhanced portion. The nodule was shown to be SNN by liver biopsy under ultrasound guidance and was followed up for 2 years with no changes in plain scan and DCE MRI.

TNM staging of PCC (UICC/AJCC, 5th Edition of TNM Staging) defined T1N0M0 in 10 lesions and T2N0M0 in 16. Distinct margins and enhanced area/nodule size >2/3 in the delayed phase were more frequently observed in cholangiocarcinoma cases than in metastatic nodules in which markedly high signal intensity (SI) on T2WI was correspondingly common (p < 0.05). Twenty-two PCCs showed sharp margins, and an enhanced area/nodule size >2/3 in the delayed phase was found in 23 (88%) cases of PCC. Only 3 PCCs appeared with regionally markedly high SI in T2WI, whereas 13 hepatic metastases presented with high SI on T2WI. Ring enhancement was the most common enhancement pattern in hepatic metastases (n = 19, 82.6%), whereas four showed heterogeneous enhancement. The results also revealed that during both the portal and delayed phases, an enhanced area/nodule size >2/3 favored cholangiocarcinoma, whereas the presence of markedly high SI on T2WI favored benign nodules (p < 0.05). Additionally, seven SNNs showed regional high SI on T2WI, most of which consisted of iso- or hypointensity on T2WI. Signs of peripheral washout on the portal or especially delayed phase only appeared in cholangiocarcinomas (n = 11, 42%; Figs 1 and 2).

**Figure 3:**
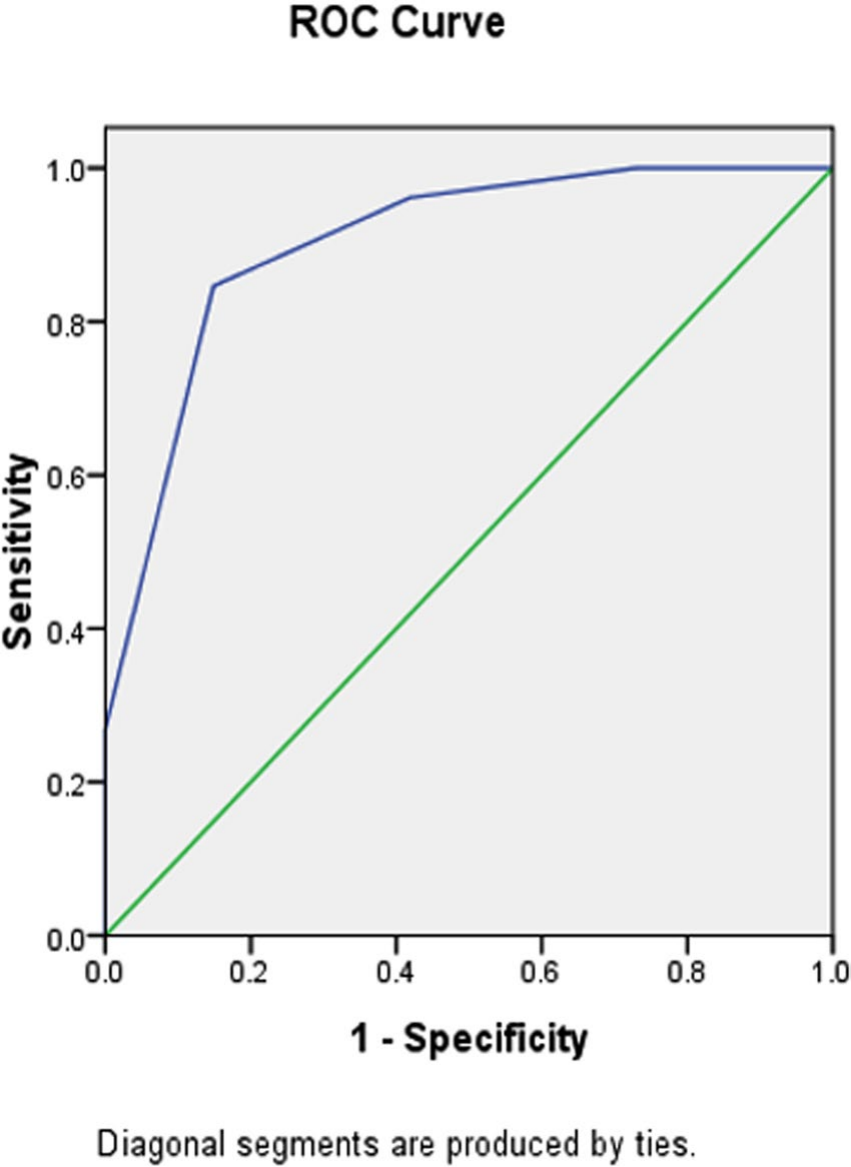
Receiver operating characteristics curves for MR diagnostic performance to differentiate PCC from non-PCC by means of statistical MR findings. The area under the curve was 0.902 ± 0.033; the sensitivity was 84.6%, and the specificity was 85.1%.

The film reading results of doctors A and B are presented in Table 3. The AUC for MRI diagnostic performance in differentiating PCC from non-PCC nodules was 0.902 ± 0.033, with 84.6% sensitivity and 85.1% specificity (Fig. 3). The *κ* values of the two doctors ranged from 0.79 to 1.00, indicating that interobserver agreement was good.

**Table 3:** Film reading results of doctor A and B.

Five-point scale^a^	PCC (26)		Non-PCC (74)	
	A	B	A	B
1	0	0	16	24
2	1	1	31	15
3	3	3	17	23
4	16	14	10	12
5	6	8	0	0

Note: a, likelihood of a PCC nodule in each patient using a five-point scale as follows: 1, definitely non-PCC; 2, probably non-PCC; 3, indeterminate; 4, probably PCC; and 5, definitely PCC. PCC, peripheral cholangiocarcinoma.

## Discussion

Cholangiocarcinoma is a primary tumor arising from the bile duct epithelium and is the second most common primary hepatobiliary cancer after hepatocellular carcinoma. At histopathologic analysis, cholangiocarcinomas are predominantly adenocarcinomas (95% of cases), although other histologic types have also been described^9^. Intrahepatic (peripheral) cholangiocarcinoma arises from beyond second-order bile ducts^10, 11^. Among the available treatment options for cholangiocarcinomas, surgery is the only curative therapy. The surgical objective for cure and long-term survival is complete tumor excision with negative histologic margins along with relief of obstruction and restoration of bilioenteric communication^12^. Thus, the treatment options depend on the site, extent, and stage of the cholangiocarcinoma. Hence, an optimal approach to the management of cholangiocarcinomas involves accurate diagnosis, characterization, localization and staging, and evaluation of adjacent structures, followed by assessment of tumor resectability and candidacy for surgical intervention or palliative therapies^13^. Imaging also plays a role in posttreatment surveillance and follow-up; however, these aspects of care are beyond the scope of this article.

Despite reports by Nanashima *et al*.^14^ and Kim *et al*.^15^ showing that 46% and 29%, respectively, of intrahepatic cholangiocarcinomas in their studies showed hypervascular enhancement patterns, peripheral nodule cholangiocarcinomas at early stages in our study presented with large areas of hypointensity at the arterial phase of dynamic enhancement.

An enhanced area/nodule size >2/3 in the portal or especially delayed phase was a common enhancement feature, which was significantly different from that of two non-PCC groups. This finding is in close agreement with previous research in which the most common enhancement pattern of cholangiocarcinoma was a peripheral rim enhancement pattern during the arterial phase followed by progressive and concentric filling with contrast material and cloud-like enhancement in delayed phases^16, 17^. The contrast enhancement pattern seems to be related to tissue composition. Although carcinoma cells proliferate in a compact pattern at the margins, they proliferate sparsely with fibrous stroma in the central area^16^.

In our case, signs of peripheral washout, which manifest as a peripheral ring-shaped area that is hypointense relative to the center of the lesion in the portal or especially delayed phase, only appeared in cholangiocarcinoma cases (n = 11, 42%). According to a previous report, the “peripheral washout sign” specifically indicates malignancy and has been described in some cases of hepatic metastases from carcinoid, breast, colon, and gastric cancers. This sign was best observed 10 min after the administration of contrast material^18^, similar to that in our study.

Owing to their high cellular water content, the majority of focal liver lesions appear hyperintense on T2-weighted images, especially malignant tumors. In a previous study, T2-weighted images were found to play an important role in distinguishing between malignant and benign lesions, particularly hemangiomas^19^. Most PCCs in early stages in our study showed slightly or moderately high signals on T2WI. Only three PCCs showed stippled high SI areas on T2WI, whereas 39 benign nodules, including 7 SNNs and 32 hepatic hemangiomas, and 13 hepatic metastases appeared to have total or only partially high SI on T2WI. In contrast to this most frequently seen MRI pattern, liver nodules may show total or partial hypointensity on T2WI. Although early stage PCCs showed almost no such performance, most SNNs in our cases had iso- or hypointensity on T2WI, which is thought to be related to a low level of hydration, vascularity, and cellularity and to the presence of coagulative necrosis^20, 21^.

Colon, lung, prostate, gastric, and transitional cell carcinomas are the most common primary tumors with hypovascular metastases to the liver. In our study, there were 12 cases of colorectal cancer, 4 cases of gastric cancer, 6 cases of pancreatic cancer and 1 case of lung cancer. These metastatic lesions usually demonstrate low SI on T1-weighted MRI and are iso- to hyperintense on T2-weighted images with ring enhancement on contrast-enhanced images^22–26^. Ring enhancement was observed consistently in hepatic metastases (n = 19, 82.6%), corresponding well with the results of Danet *et al*.^22^.

This study had several limitations. First, because it was a retrospective study, some inherent selection bias was present. Second, the study was limited to hepatic solitary nodules, making the sample size relatively small. Third, a precise correlation between the imaging findings and the overall histological composition was not performed. Last, surgical correlations were not available for all our patients. Most cases of hepatic hemangiomas required no further invasive procedures or surgery. However, in these cases, we had a firm clinical diagnosis and imaging follow-up.

In conclusion, a distinct margin, an enhanced area/nodule size >2/3 in the portal or especially delayed phase, and the “peripheral washout sign” in the delayed phase may facilitate differential diagnosis because these features are significantly different in cholangiocarcinomas of stages T1N0M0 and T2N0M0 in comparison to other hypovascular nodules (≤3 cm).

## Methods

### Patients

This retrospective study was approved by the Institutional Review Board of Huadong Hospital Affiliated to Fudan University, and informed consent was waived. Between January 2005 and August 2014, liver MRI examinations documented in the radiological database, including both unenhanced and 3D dynamic contrast-enhanced (DCE) imaging, were reviewed.

The following study inclusion criteria were applied: (a) patients with pre-contrast and contrast agent enhancement at multiple phases (e.g., no contrast enhancement, arterial phase, portal venous phase, delayed venous phase and even ultra-delayed venous phase), (b) solitary hepatic lesions, (c) maximum diameter of any hepatic nodules ≤3 cm, and (d) hypovascular nodules, which were defined as nodules with large areas of hypointensity in the arterial phase of dynamic enhancement.

Exclusion criteria consisted of patients histopathologically proven to have dysplastic nodules, hypovascular well-differentiated hepatocellular carcinomas, simple or complex cysts, and other uncommon hypovascular nodules such as angioleiomyolipomas.

One hundred patients were included in the final study population (67 men, 33 women; age range, 27–91 years; mean age, 54 ± 11 years), including 26 peripheral nodular cholangiocarcinomas (PCCs; 17 men, 9 women; age range, 37–71 years; mean age, 57 ± 9 years), 23 HMs (15 men, 8 women; age range, 41–69 years; mean age, 56 ± 9 years), 19 SNNs (11 men, 8 women; age range, 42–74 years; mean age, 58 ± 8 years), and 32 atypical HGs (24 men, 8 women; age range, 27–91 years; mean age, 47 ± 13 years). All peripheral nodular cholangiocarcinomas were confirmed by surgery (n = 23) and biopsy (n = 3); of these patients, 18 had abdominal discomfort, 2 showed weight loss, and 6 were asymptomatic. Twenty-three metastases were confirmed by surgery (n = 19) or biopsy (n = 4); all 23 cases appeared asymptomatic and were detected by follow-up imaging, with 12 showing colorectal cancer, 4 with gastric cancer, 6 with pancreatic cancer and 1 with lung cancer. Nineteen SNNs were confirmed by surgery (n = 19); of these patients, 6 had abdominal discomfort, and 13 were asymptomatic. All atypical hepatic hemangiomas, which were confirmed by biopsy (n = 8) or segmentectomy (n = 4) and follow-up imaging (n = 20), were asymptomatic. The longest follow-up time was 5 years.

### MRI examination

MRI was performed using a 3.0 T scanner (Magnetom Trio, Siemens, Erlangen, Germany) with an 8-channel body-phased array coil. The following baseline MRI images were acquired with the following parameters: 1) breath-hold T1-weighted GRE sequence: repetition time (TR), 139 ms; echo time (TE), 2.46 ms; flip angle, 66°; matrix, 320 × 256; average, 1; section thickness, 6 mm; distance factor, 20%; and acquisition time, 20 s: 2) navigator-triggered, fat-suppressed, and T2-weighted turbo spin echo sequence with the PACE technique: TR (effective), 4,781 ms; TE (effective), 81 ms; flip angle, 140°; echo train length, 12; matrix, 256 × 256; number of signals acquired, 1; section thickness, 7 mm; distance factor, 20%; and acquisition time, approximately 90 s; 3) T2-weighted half-Fourier acquisition single-shot turbo spin echo sequence: TR/TE, 4,500/78 ms; 150° flip angle; matrix, 256 × 256; and slice thickness, 6 mm; and 4) breath-hold T1-weighted fast low angle shot sequence: repetition time, 172 ms; time to echo, 2.50 [in-phase]/1.22 ms [out-of-phase]; 65° flip angle; matrix, 208 × 256; signal average, 1 and 2 acquisitions; and slice thickness, 5 mm. Dynamic imaging with a three-dimensional volumetric interpolated breath-hold examination (3D-VIBE) sequence was obtained before (pre-contrast) and 20 s (arterial phase), 50 s (portal venous phases), and 3–5 min (delayed phases) after intravenous administration of Gd-DTPA (Magnevist; Bayer Schering Pharma). Ultra-long time-delayed phase acquisitions were performed at 10 min. All images were acquired in the transverse plane with a field of view of 370 mm to cover the entire liver. Gd-DTPA was injected intravenously as a bolus (3.0 ml/s) at a dose of 0.1 mmol/kg of body weight followed by 20 ml of a saline flush using a power injector. The average interval between MRI and surgery was 10 days (range, 1–30 days).

### Image interpretation

Two radiologists with 10 years of clinical experience in abdominal MRI independently analyzed all images and described the imaging characteristics. Four weeks later, the two radiologists, blinded to the pathologic and clinical findings but aware of the statistical results, assessed the likelihood of the nodules being benign or malignant. Combined unenhanced and contrast-enhanced MRI images were randomly assigned to each observer. Each observer independently and separately interpreted the MRI images. All MRI images were evaluated on picture archiving and communication system monitors. Any patients whose images did not meet the image quality control were removed from this study.

All analyses were conducted at the nodule level (≤3 cm), assessing the relationship between location, margin (defined or ill-defined edge on trigged), regionally markedly high SI on T2WI, enhanced area/nodule size in the portal and delayed phases, and the presence of a peripheral ring-shaped weak enhancement area. The long diameter of each mass was also measured. The reviewers rated the likelihood of a PCC nodule in each patient using a five-point scale as follows: 1, definitely non-PCC; 2, probably non-PCC; 3, indeter+minate; 4, probably PCC; and 5, definitely PCC.

### Statistical analysis

Chi-square test or Fisher’s exact test and multivariate analyses were used to compare MRI findings. These findings were used to differentiate PCC from non-PCC nodules. Diagnostic performance for differentiating PCC from non-PCC lesions was then demonstrated using area under the receiver operating characteristic (ROC) curve (AUC). A difference with p < 0.05 was considered statistically significant for all tests. IBM SPSS version 20.0 software (IBM SPSS, Inc., Chicago, IL, USA) was used for statistical analysis.

## Electronic supplementary material

Supplementary Dataset 1
